# Perception of saccadic reaction time

**DOI:** 10.1038/s41598-020-72659-3

**Published:** 2020-10-14

**Authors:** Valentina Vencato, Laurent Madelain

**Affiliations:** 1 UMR 9193-SCALab, CNRS, Univ. Lille, 59000 Lille, France; 2 Institut de Neurosciences de la Timone, UMR 7289, CNRS, Faculté de Médecine de la Timone, Aix Marseille Université, 27 Bd Jean Moulin, Marseille, 13005 France

**Keywords:** Saccades, Decision, Perception, Motor control, Reward

## Abstract

That saccadic reaction times (SRTs) may depend on reinforcement contingencies has been repeatedly demonstrated. It follows that one must be able to discriminate one’s latencies to adequately assign credit to one’s actions, which is to connect behaviour to its consequence. To quantify the ability to perceive one’s SRT, we used an adaptive procedure to train sixteen participants in a stepping visual target saccade paradigm. Subsequently, we measured their RTs perceptual threshold at 75% in a conventional constant stimuli procedure. For each trial, observers had to saccade to a stepping target. Then, in a 2-AFC task, they had to choose one value representing the actual SRT, while the other value proportionally differed from the actual SRT. The relative difference between the two alternatives was computed by either adding or subtracting from the actual SRT a percent-difference value randomly chosen among a fixed set. Feedback signalling the correct choice was provided after each response. Overall, our results showed that the 75% SRT perceptual threshold averaged 23% (about 40 ms). The ability to discriminate small SRT differences provides support for the possibility that the credit assignment problem may be solved even for short reaction times.

## Introduction

Our environment is constantly changing and we need to gather new sensory information to successfully interact with it. However, much as other resources, information is not evenly distributed across space or time. Hence, animals must learn to adjust their information-foraging behaviour in response to the specific spatiotemporal organization of their environment. This necessity is particularly striking for visual information because, in foveate animals, only a fraction of the visual surroundings can be monitored at any given moment, and it is thought that the need to orient toward visual stimuli at the right time provides strong evolutionary pressures that might shape their eye movement systems^1–3^. The ability to learn to adapt the spatial allocation of visual resources depending on environmental constraints has been repeatedly demonstrated in the primate saccadic-system, for instance by inducing changes in the direction or amplitude of saccades in response to the experimental manipulation of the post-saccadic position-error signal^4–6^. The temporal plasticity of saccade triggering has also been well established: saccade reaction time distributions have been shown to change in response to instruction^7^, perceptual task or motivation^8–10^, predictability of the timing of target onset^11,12^ or reinforcement contingencies^13,14^. It is noteworthy that these last results are nicely in line with previous works demonstrating the adjustment of inter-response-time distributions to reinforcement contingencies in other modalities^15–17^.

That humans might learn to adjust the timing of their eye movements according to environmental regularities has been elegantly quantified in a study^18^ in which participants were required to detect the appearance of a dot at one of two possible locations. When the probability of stimulus appearance at each location was manipulated, participants were able to quickly adjust the temporal properties of their visual exploration to efficiently monitor the visual space and detect the event. In the same vein, a recent study^14^ showed that when reinforcement is contingent upon specific saccadic latencies in a choice paradigm, humans learned to adjust their SRT distributions depending on the reinforcement contingencies in force: the relative proportions of short- and long-latency saccades matched the relative proportions of reinforcement obtained for each class of latencies. Eye movement studies outside the laboratory with sportsmen^19^ or video-gamers^20^ demonstrated similar temporal-learning abilities. It is worth noting that low-level target properties, such as luminance, have been shown to compete with internal factors based on previous experience in controlling saccade reaction time^9,21–23^. Because saccade duration is not actively controlled but depends on the movement amplitude^24^ controlling saccade latencies is necessary to control when the eye lands. This is not the case for other movements, such as reaching movements, in which the movement duration is actively controlled to correct for temporal errors^25^. Overall, these researches revealed that the temporal organization of the environment constrains the temporal allocation of saccades.

To adapt saccade triggering to learned temporal regularities of dynamic environments the saccadic system must not only have the ability to exert some control over when to saccade but also have some knowledge about its own reaction time. Indeed, when receiving a reinforcer in a latency-contingent saccade paradigm, the saccadic system needs to connect the cause, i.e. the specific latency, and the consequence, i.e. the occurrence of an event defined by the reinforcement contingencies. In other words, the saccadic system must solve the assignment-of-credit problem, that is the ability to extract what in the behavior caused the appearance of the reinforcer^26–28^. In supervised-learning situations an unambiguous error information is presented to the learning system and assignment-of-credit is explicitly solved by the supervisor. This is the case, for instance, in conventional motor-adaptation experiments in which the post-movement distance to the target provides an unambiguous spatial error-signal, or in interception tasks in which this distance provides temporal error-signals. These signals are thought to drive movement adaptation^29,30^. However, experiments demonstrating plasticity in saccade reaction time (SRT) distributions have been conducted in unsupervised learning paradigms in which the presence or the absence of a reinforcing event is the only signal available to the learning system (e.g.^8,13,14,31^). It is noteworthy that, although many studies explored the effects of experimental manipulations on the production of SRTs^32^, the extent of SRT perception has not been investigated so far. We argue that both SRTs production and perception must be quantified to describe the underlying mechanisms controlling saccade triggering.

In spite of the large literature on the psychophysics of time perception, quantifying the extent of SRT perception poses some specific methodological challenges. Indeed, several paradigms have been conventionally used to quantify the extent of time perception, often relying on temporal interval comparison (either using a single stimulus or a sequence of intervals) or interval reproduction^33,34^. In these tasks a sample interval is delivered, e.g. using a visual or an auditory stimulus, to the participant who is asked to either reproduce it or estimate its duration in relation to a comparison interval (or two anchor intervals in the case of the bisection task). These types of paradigms are not well suited to quantify SRT perception. One fundamental issue with paradigms relying on reproduction is that variability in the produced intervals may originate from both noisy perception and a noisy motor control (see^35^ for a similar concern). Combining these two sources of noise is likely to considerably affect the precision of the temporal perception estimations for short durations, typical saccade latency in reaction to the appearance of a visual target often lasting about 150 to 200 ms. Second, in the case of SRTs, the duration to be perceived is produced by the participant himself, not by the experimenter. This is problematic because participants might knowingly produce shorter or longer latencies to facilitate the comparison. Indeed, in an unpublished study, we asked participants to classify their SRTs according to four categories (very short, short, long, and very long) based on the quartiles of their individual SRTs baseline distribution. We observed gradual shifts in SRTs distribution, suggesting that participants strategically controlled their reaction time when performing the psychophysical task. This cast doubts on the outcome of comparison-based paradigms in which observers are likely to choose using the intended latency rather than the perceived one.

To avoid the potential pitfalls of these conventional time perception paradigms, we resolved to ask observers to report the perceived absolute durations of their SRTs using a two-alternative forced choice task in which the two options were the actual saccade latency expressed in milliseconds and an incorrect value that was proportional to the actual latency. The incorrect option was computed on a trial-to-trial basis by adding or subtracting a given percent-difference to the actual latency. For instance, for a 200 ms saccade latency the two options would have been 200 and 250 (or 200 and 150) for a 25% percent-difference or 200 and 210 (or 200 and 190) for a 5% percent-difference. Along the course of the experiments, we were therefore able to adjust the magnitude of the distance between the correct and the incorrect option, hence the difficulty of the choice task, using a family of these percent-differences (ranging from 2.5 to 50%). This allowed constructing psychometric functions estimating the perceived duration of SRTs. The choice to use a percentage for the difference between the two options was supported by the fact that it has been established that time perception follows the scalar property, i.e. that variability in duration perception linearly increases with the mean temporal interval^34^. This paradigm shares some properties with the verbal estimation of duration paradigms in which the observer is asked to report the perceived duration of stimuli using conventional time units (typically seconds) rather than comparing two durations. However, these measurements usually use several temporal intervals as samples that typically range from 250 to 2000ms^33^. In our case, the range of temporal duration was much narrower as saccade latency in response to a single target onset typically ranges from 100 to about 300 ms, durations that are rarely verbally reported in daily life. We, therefore, resolved to systematically provide feedbacks signaling the correct choices to help observers calibrating their answers based on the perceived durations. Importantly, previous studies have shown that such feedbacks do not significantly affect the averaged perceived durations but might reduce the variability in choices^36^.

The experimental procedure included two phases: a learning-phase followed by a testing-phase. In the learning-phase, we trained participants to estimate their SRTs using an adaptive procedure in which the percent-difference decreased following correct choices (all details of the learning phase are in Supplementary Information). Analysis of the performance during these learning sessions allowed us to estimate the just-noticeable-difference. In the testing-phase, a method of constant stimuli was implemented to estimate the 75% threshold of duration perception by fitting a psychometric function (LD task, Experiment 1.1). In this phase, the specific percent-difference used in a given trial was randomly selected among a set of 20 percent-difference ranging from 2.5 to 50%.

Three control experiments have been implemented. First, we asked observers to perform the exact same psychophysical task as in the main experiment but in the absence of a temporal duration to be estimated (DT task). In this control task the participant was not required to do a saccade, so no SRT had to be estimated. Observers had to choose one of two options (where one was based on actual, previously collected, SRTs) and received feedbacks signaling the correct option. We hypothesized that choice could have been biased by using an estimated mean of their SRT distribution around which the correct value is distributed. Second, we estimated the time perception of the duration of a visual stimulus, using the same choice paradigm. Finally, we estimated the time perception of the duration of an auditory stimulus, again using the same choice paradigm. Indeed, it has been established that observers judge the duration of auditory stimuli more precisely than stimuli presented in other modalities^37–41^. However, the difference in performance across modalities decreases for smaller intervals.

## Results

### Latency duration perception task (LD)

Participants were asked to estimate their saccade latency in a visually guided saccade task. After each valid saccade, two numbers were displayed on the screen, one indicating the actual saccade latency in milliseconds and one an incorrect value—which corresponds to the actual saccade latency plus or minus a fraction of the latency, a percent-difference. For instance, for a 200 ms latency saccade and a percent-difference of 25%, the participant would have to choose either between 200 and 250 (200 + 25%) or between 200 and 150 (200–25%). Participants were instructed to choose the value corresponding to the perceived saccade-latency duration and a feedback was given to signal a correct or incorrect choice. In the learning-phase (seven 200-trial sessions), the percent-difference used for the incorrect value was set using an adaptive procedure so that the resulting distance between the correct and incorrect options would progressively decrease following correct choices (details of this learning phase are in Supplementary Information). In the testing phase (three 220-trial sessions), we used a method of constant stimuli in which the percent-difference used for the incorrect option was randomly selected within a set of 20 percentages (ranging from 50 to 2.5%) and either added or subtracted to the latency value in order to compute the incorrect option. Individual perceptual thresholds were then estimated by fitting the data obtained in this testing phase to obtain a psychometric function (see “Methods”).

**Figure 1:**
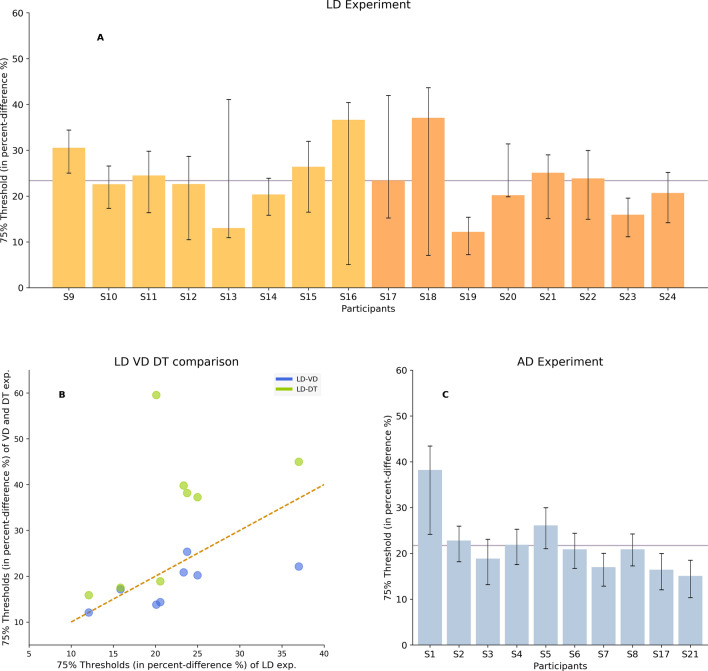
(**A**) 75% perceptual thresholds in the LD experiment of the first 8 participants (light orange) and of the further 8 participants for the replication of the results (dark orange). Black error bars plot the 95% confidence intervals. (**B**) 75% perceptual thresholds in VD (blue) and DT (green) experiments plotted as a function of 75% perceptual thresholds in LD experiment. The red dashed line shows equal values. (**C**) 75% perceptual thresholds in the AD experiment of 10 participants. Black error bars plot the 95% confidence intervals. The horizontal line plots the average threshold across participants.

We first obtained 75% perceptual thresholds in 8 participants (S9–S16, light orange, Fig. 1A). Perceptual thresholds—expressed in proportion of the saccade latencies—averaged 24.5% and were quite variable across participants, ranging from 13 to 36.5%. For these participants, saccade latencies averaged 172 ms, ranging from 138 to 217 ms. These values can be used to estimate the absolute 75% perceptual thresholds expressed in milliseconds using the individual median latencies: We found that the thresholds corresponded to an average duration of 42 ms. Using the same protocol, we replicated these results in 8 other participants (S18–S24, dark orange, Fig. 1A) who participated in two control experiments as well (see below). Perceptual thresholds were similar in this second group, averaging 22%, as were the saccade latencies with an average of 175 ms.

Pooling our 16 participants together the 75% perceptual thresholds averaged 23%, ranging from 12 to 37%. Expressed in milliseconds the thresholds averaged 40 ms, ranging from 19 to 63 ms. We computed the coefficients of variation of the perceptual thresholds expressed in percent (0.30, 90% CIs [0.20; 0.37]) and in milliseconds (0.32, 90% CIs [0.22; 0.39]). The difference between the two coefficients of variation was not significant (0.02, smaller than the null hypothesis 98% Cis, 0.15, estimated by Fisher’s exact test with 100,000 permutations), revealing a similar consistency across participants. Because our participants were asked to report the perceived duration of their own saccade latencies we had no direct control over the actual duration to be estimated. It is, therefore, possible that the individual latency distributions influenced the measured thresholds. However, our data did not reveal a significant correlation between the estimated thresholds and the median latencies (p-value = 0.58). This is to be expected as we used proportions of the actual latency to quantify the thresholds. Moreover, the correlation between the perceptual thresholds and the latency dispersion quantified using the inter-quartile range, i.e. the difference between the first and last quartile of the latency distribution, was not significant as well (p-value = 0.62), indicating that the perception of latency might be relatively immune to the latency variability. Finally, we asked whether the discrimination was different for latencies falling within the most central percentile bin of the distribution when compared to two more extreme latencies. Thus, the threshold of each participant has been calculated in three bins of the latency distribution. The criterion used to select the bins was decided for the purpose of having 33% of the data in the first bin (from the 1st to the 33th percentile), 33% of the data in the central bin (from the 33th to the 66th percentile), and 34% of the data in the third bin (from the 66th percentile to the 100th percentile). A Kruskal–Wallis test has been performed to assess the difference among thresholds median across participants in the three bins. The results showed no significant difference among the medians of the three bins (p-value = 0.09). The Wilcoxon sign-rank also showed no statistical difference between the central bin and the first one (p-value = 0.08) and between the central bin and the third one (0.06). That we did not find any systematic facilitation of discrimination for latencies closer to the median distribution does support the idea that participants did not respond by simply estimating the central tendency of their saccade latency distributions but instead perceived the actual duration of the time elapsed between the visual target- and saccade-onset. A control experiment was implemented in the same subjects in order to quantify the contribution of this potential distribution estimation on the measured thresholds.

### Control experiment 1: distribution estimation task (DT)

We designed a task in which 8 of our participants were asked to perform the same choices as the ones in the LD task but in the absence of an actual duration to be estimated. We reasoned that, if participants only used an estimate of the central tendency of the latency distribution to orient their trial-by-trial choices, measured thresholds should be identical when relying solely on the numerical values previously signaled as correct. Participants were therefore asked to perform a two-alternative forced choice between the same pairs of options they encountered in the LD task but in the absence of a saccadic movement. The instructions stated that: “One out of the two numbers appearing on the screen, is the correct number; we cannot tell on what basis this is correct but a feedback is provided”. In a first learning-phase, we ran three 200-trial sessions to familiarize participants with the task while in a second testing phase three 220-trial sessions to quantify the threshold (75%) using the constant stimuli method. The pairs of options presented in both the familiarization and the testing phase were chosen amongst the pairs of options presented in the corresponding session learning-phase (and keeping the same order such that choice difficulty increased during the session) and testing phase (in random order) in the LD experiment, respectively. Therefore, one of the two numerical options represented an actual saccade latency, i.e. a value reflecting the duration of one saccade amongst the entire distribution previously collected. Participants were instructed to choose one of the two values and correct choices were signaled by a visual feedback (the correct value turned green) as in the LD task. The estimated 75% thresholds averaged 34% (Fig. 1B), ranging from 16 to 60%. Importantly, thresholds measured in this task were systematically higher than when participants had access to an actual latency duration (green dots, Fig. 1B) with a difference averaging + 12%, except for one subject (S24) in whom the thresholds were similar. To test the hypothesis that the sample thresholds in LD and DT experiment are drawn from the same distribution (H_0_) a Wilcoxon signed-rank test to assess the medians difference has been conducted. Results showed that the two medians were significantly different (p-value = 0.02). In addition, correlation between LD and DT thresholds was not significant (p = 0.15).

This indicates that subjects did not rely solely on an estimation of the saccade latency distributions but instead used the actual latency duration in the LD task.

### Control experiment 2: visual stimulus duration task (VD)

In our saccade task, the saccade latency interval is perfectly correlated with the duration of the visual signal related to the presence of the saccade-target in the periphery. One could therefore use the duration of this visual signal as a proxy to estimate the SRT. To probe whether purely visual signals might be used to estimate saccade latencies, the same eight participants were asked to report the duration of a visual stimulus appearing at the center of the screen. Visual stimulus duration corresponded to the saccade latency durations in the equivalent session of the same participant in the LD task. The values were rounded to the nearest 10 ms as our screen refresh rate was set at 100 Hz. Immediately following the visual stimulus presentation, two numbers appeared and participants had to choose the option corresponding to actual visual stimulus duration in milliseconds. Six 200-trial sessions were first performed in a learning-phase followed by three 220-trial sessions in a testing phase. The estimated 75% thresholds averaged 18% (blue dots, Fig. 1B), ranging from 12 to 25%. Although thresholds in this task were on average lower than when estimating saccade latencies by 4%, this tendency was not systematic as it was true in 5 participants but not in the other 3. The correlation between LD and VD thresholds was not significant (p = 0.07). LD and VD tasks medians difference were assessed by using the Wilcoxon signed-rank test and no significant difference was found (p-value = 0.08). Importantly, estimating the actual duration of the visual stimulus systematically lead to a lower threshold than when participants have no access to a temporal signal (DT task), with a difference averaging + 15% (p-value = 0.008). Again, this indicates that subjects did not rely solely on an estimation of the distributions of the values but instead were able to perceive the actual temporal duration of the visual signal.

### Control experiment 3: auditory duration task (AD)

To probe whether the threshold we measured when using saccade latencies were specific to these type of temporal signals 10 participants performed an additional task in which they were instructed to report the duration of an auditory stimulus. A sound was presented through headphones and its duration was randomly chosen among a set of values distributed according to bell-shaped non-parametric curve with the median around 205 ms and interquartile difference of around 219 ms. Immediately following the auditory stimulus presentation, two numbers appeared on the screen and participant had to choose the option corresponding to the actual sound duration in milliseconds, as in the other experiments. Six 200-trial sessions were first performed in a learning-phase followed by three 220-trial sessions in a testing phase. The estimated 75% thresholds averaged 21% (Fig. 1C), ranging from 15 to 38%. No significant difference was found when comparing the thresholds measured when estimating saccade latencies to the ones measured when estimating the sound duration (p-value = 0.44).

### Effects of the feedback on discrimination performance

It has been shown that perceptual history can bias the current perception^42^. To probe the effect of feedback on participants’ performance we investigated two possible strategies that observers may have used in the LD task: the tendency of responding according to either the correct (signaled) value in the previous trial or an estimation of the mean value of all signaled correct responses.

In the first case, an index has been computed to weight the bias toward or away from the correct response in the previous trial for incorrect choices. Specifically, the index varies from − 1 to 1; a negative index denoting a tendency of choosing the option that differed more from the correct value in the previous trial compared to the other option while a positive index signals a tendency of choosing the option closest to the correct value in the previous trial compared to the alternative option. Values around 0 represent a weak or no tendency to the bias.

Seemingly, a bias index has been computed to quantify the tendency of choosing according to an estimated mean latency across the session. Namely, previous latencies could have been averaged to predict the future correct options. The estimated mean was obtained by averaging all chosen values in all trials in every session (whether correct or wrong). The bias index was computed by adding 1 each time that the value chosen in one specific trial was the one closer to the estimated mean. On the contrary, 1 was subtracted when the chosen value was the one far from the estimated mean. The number obtained was then divided by the total amount of the wrong responses in the session. The index scales from − 1 to 1, where negative values reveal a tendency to choose options far from the estimated mean and, on the opposite, a positive index reveals a tendency to choose the option closest to the estimated mean. Again, values around 0 represent a weak or no tendency to the bias.

The index obtained in the LD task has been then compared to the same index computed in VD and DT tasks. For the LD-VD comparison, we expected a systematically larger bias due to the possible larger perceptual noise associated with latencies. We reasoned that if SRTs perception is more difficult than the visual duration estimation, participants should rely more on that strategy in LD than in VD and vice versa. In particular, we expect that visual stimulus should provide a more reliable signal such that the bias in the VD task should be systematically smaller. In the LD-DT comparisons we expected larger biases in the DT task than in the LD task, as the only source of information that participants can use in order to perform the task is provided by the feedbacks.

A correlation between the perceptual thresholds at 75% and the bias index has been computed in both kinds of bias and in all experiment.

**Figure 2:**
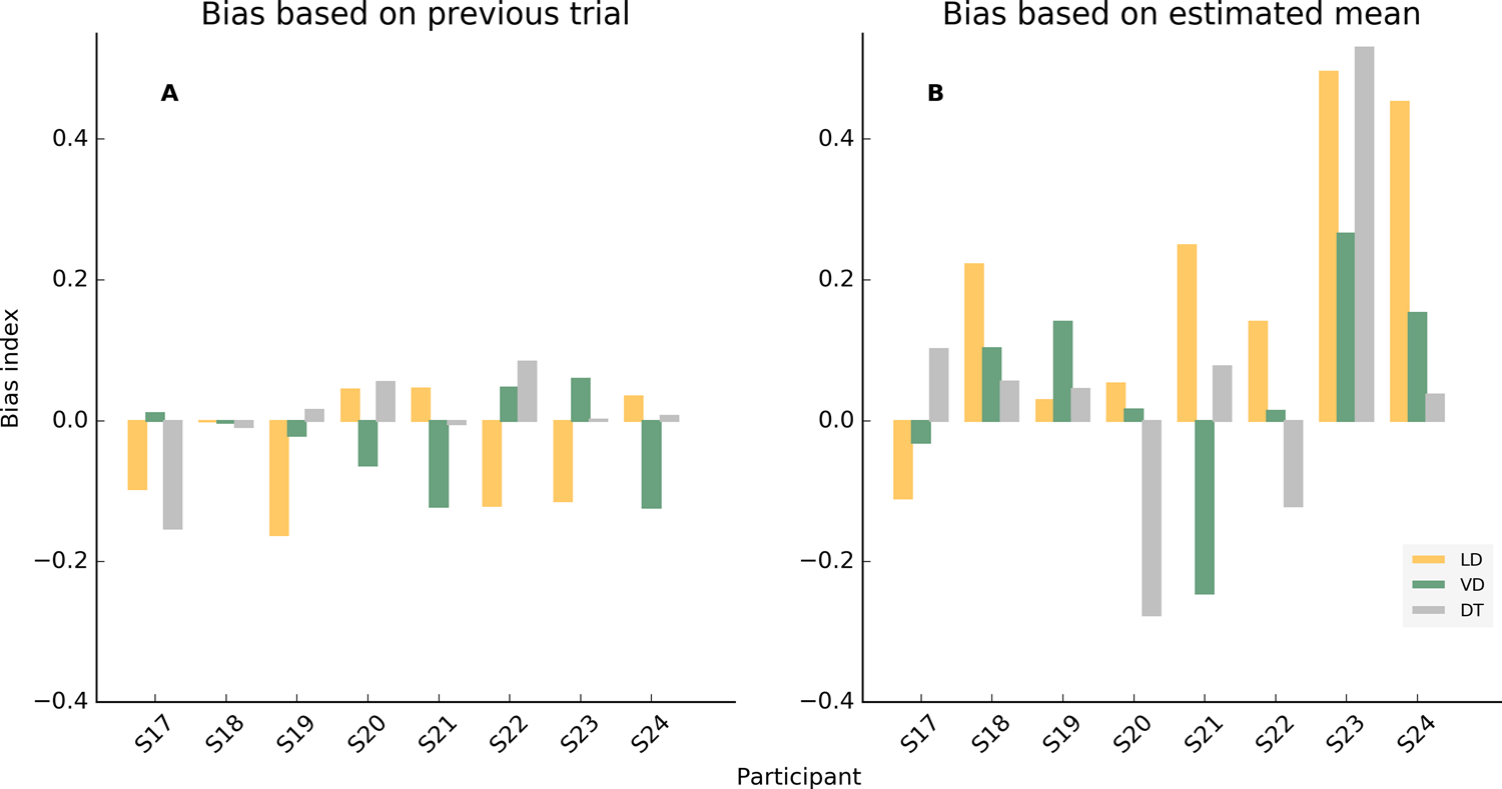
(**A**) Bias index based on the preceding trial in the LD (orange), VD (green) and DT (grey) experiments for each participant. Positive values indicate a bias toward the value closer to the correct one in the previous trial, negative values indicate the opposite tendency. (**B**) Bias index based on the overall mean duration estimate in the LD (orange), VD (green) and DT (grey) experiments for each participant. Positive values indicate a bias toward the estimated mean, negative values indicate the opposite tendency.

Bias based on the previous trial did not reveal any systematic pattern across participants. Index values ranged from − 0.16 to 0.08 (Fig. 2A). Correlations between the 75% perceptual thresholds and bias index in LD, VD, and DT were not significant; the p-values are respectively: 0.25, 0.31, 0.92.

Bias based on the estimated mean (Fig. 2B) ranged from − 0.11 to 0.54 across participants in the LD task (mean equal to 0.18), from − 0.25 to 0.27 in the VD task (mean equal to 0.05), and from − 0.28 to 0.53 in the DT task (mean equal to 0.11). Again, no systematic pattern was found across participants. In LD-VD comparison participants S17, S18, S22, S23, and S24 tended to have a lower bias in VD than in LD, even though two of them seemed to use very different strategies (S17 and S21). Bias in the DT task was not consistent across participants. Correlations between perceptual thresholds at 75% and the bias indexes in LD, VD and DT tasks were not significant (p-values 0.96, 0.35 and 0.08 respectively).

## Discussion

**Figure 3:**
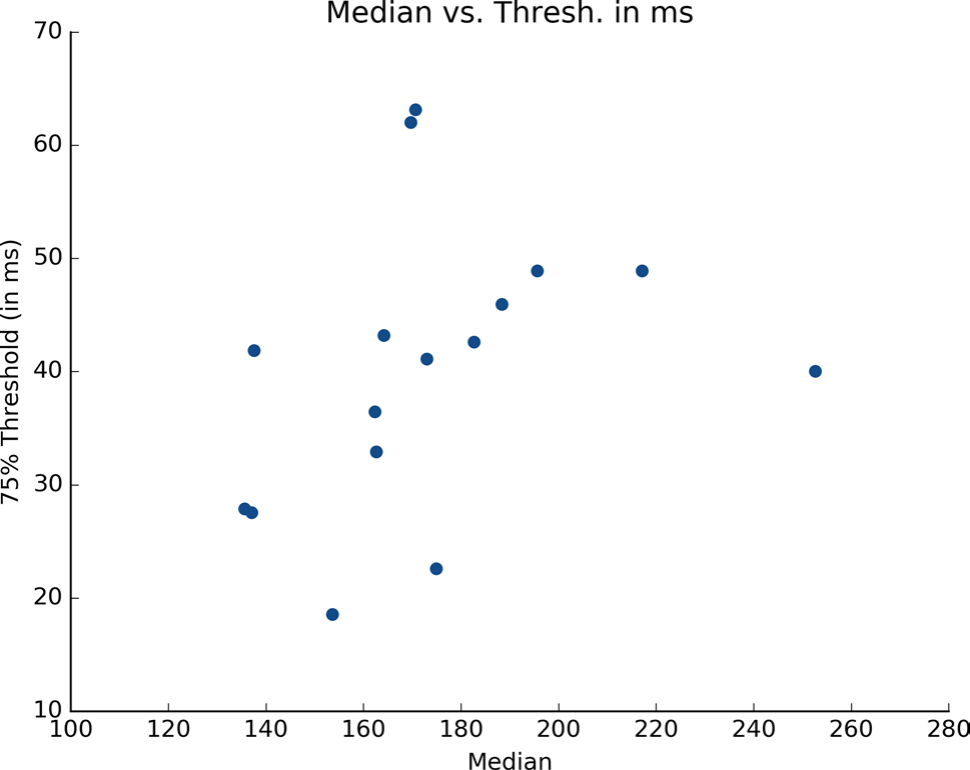
Perceptual 75% threshold as a function of the median saccade latency for each participant.

In the current study, we investigated the extent of perception of the duration of saccadic reaction time by human observers. After training, we found 75% discrimination thresholds as low as 12% (participant 19). This implies that we estimate her absolute threshold to be as low as 18 ms as the median latency distribution for this particular participant was 154 ms. On the other hand, the highest estimated threshold reaches 37% (participant 18) which, with a median latency reaching 171 ms, indicates an absolute threshold of 63 ms. Across our observers, we estimated that the average absolute 75% threshold was about 40 ms (Fig. 3). It is noteworthy that another set of experiments recently demonstrated that participants were able to produce two classes of SRTs, i.e. short and long, in response to specific reinforcement contingencies^14^. In these experiments, the authors used the first and last quartiles of the individual SRT distributions to construct the two classes of latencies to be produced. For instance, for one particular participant, latencies between 80 and 152 ms were categorized as short, and latencies ranging from 185 to 300 ms were categorized as long such that the distance between the two latencies classes was 33 ms. Across participants, the average distance between the two classes was 37 ms. The 40 ms estimated average threshold is therefore in agreement with these results as it indicates that human observers are able to perceive the difference between such two classes of SRTs. Considering these results together, we argue that the saccadic system may use the perceived latency duration to solve the assignment-of-credit problem and adapt its reaction time to specific environmental contingencies.

In SRT learning experiments adaptation is certainly consistent across subjects but with strong intra-individual variability^8,13,14,31^. In these situations, three sources of uncertainty may be viewed as controlling the individual performance: the ongoing contingency^43,44^, the SRT production itself^32^, and, as we argued before^14^, the perception of time. The systematic intra-individual variability in SRTs has been well established before^45^, even in situations in which participants experienced long learning phases with unchanged contingencies (e.g.^13,14^). Accounting for this motor variability has been instrumental in shaping the current computational models of saccade triggering (e.g. Ratcliff’s drift model^46,47^ or Carpenter’s LATER model^48^) in which a noisy decision process is modulated by endogenous factors. In the present experiment, the average 75% perceptual threshold measured for saccade latencies reached 23%, a value that, on the one hand, reveals the general ability to perceive SRTs but, on the other hand, indicates that this perception is quite variable across trials. Overall, our present results support the possibility that intra-individual variability in SRT control may be, at least partly, linked to the imperfect perception of reaction time.

The exact nature of the signal used to perceive saccade latency remains obscure. Indeed, two types of intervals have been operationalized in time perception experiments as filled versus unfilled (or empty) intervals and research have shown that perception is systematically more accurate when using filled intervals—in particular when a single presentation is used^49–51^. Our LD paradigm does not disentangle between these two types of intervals. On the one hand, one could argue that saccade latency intervals are marked by two discrete events, (i) the saccade-target onset at the beginning of the interval and (ii) either a change in the visual signal or a proprioceptive signal at saccade onset marking the end of the interval. Therefore, the temporal interval might be regarded as an unfilled interval. On the other hand, one may argue that the duration of the visual signal conveyed by the presence of the saccade-target in the periphery is a perfect proxy for the saccade latency duration. If it were the case, estimating saccade latency would be strictly equivalent to estimating the duration of a visual stimulus marking a filled interval. Results from our Visual stimulus duration task are compatible with this possibility: despite a lower average perceptual threshold, individual performances were not systematically better when compared to the latency estimation task. It is also noteworthy that the perceptual thresholds estimated using an auditory signal were comparable to the ones obtained with a visual stimulus, a result that has been reported before when using short filled intervals^52,53^. Although we assumed that SRTs were treated as filled intervals we cannot exclude the possibility that some—or all—observers perceive it as empty intervals which might contribute to a higher threshold and larger variability. Further experimental studies are necessary to disentangle between these two possibilities. For instance, flashing the saccade-target, instead of displaying it during the whole saccade latency interval, would ensure the perception of unfilled durations.

Because of the specificity of the temporal intervals that observers had to estimate and based on pilot experiments we resolved to implement a learning-phase and to give feedback on every trial signaling the correct option. One could argue that this feedback changes our task as subjects could attempt to use an estimate the mean of the SRT distribution to perform the 2-AFC task rather than using the perceived duration of the temporal intervals. There are however several reasons to reject this possibility. First, this hypothesis suggests that the variability in SRT should impact the performance of the observer but there was no correlation between the dispersion in the SRT distributions and the estimated 75% perceptual threshold. Second, observers should give more correct answers for SRTs falling within the central part of the SRT distribution (i.e. comprised within the first and third quartile), but our data revealed that this was not true for half of the observers. Third, when observers were asked to choose between the two options in the absence of saccade latencies in the DT task, the measured thresholds were on average higher (34% vs. 23% in the saccade task) and this was true at the individual level as well for all but one participant. Fourth, thresholds when estimating the SRT duration were comparable to the ones obtained when using intervals based on the duration of a visual or auditory signal, a result which supports the notion that the true duration of the intervals was used to respond in all three experiments. Finally, we quantified a bias index in all experiments to estimate whether observers responded using an estimated SRT mean. In the LD task the bias was overall small (averaging 0.18) but quite variable across participants. In addition, the average value of the bias indexes in the LD task was larger than the one in VD (0.05) and DT task (0.11). Nevertheless, there is no relationship between the performance and the bias. Indeed, no significant correlation has been found between the perceptual 75% threshold and the bias index in the LD task. On the contrary, in the DT experiment, the tendency of a negative correlation between threshold and bias has been found. This may suggest that, on the one hand, our index may be a good predictor of the mean estimation strategy used by the participants (indeed, using this strategy was the only way to perform in DT task). On the other hand, it indicates that in the LD task, performances cannot be solely accounted for by this strategy. Furthermore, in our task, there might a possible confound due to the central tendency effect^54,55^. This ‘regression’ toward the mean value may be explained by this well-established effect in time perception in which relative long durations tend to be underestimated while relative short durations tend to be overestimated.

It has been shown that time perception may be distorted by saccades, an effect present right before and after a saccade as well as during its execution^56,57^: time appears compressed and observers report shorter durations when the duration to be estimated overlaps with a saccade. This time illusion persists during a saccade planning even in those cases in which the movement is suppressed^58^. Although this phenomenon has been demonstrated when judging the duration of a stimulus it could be that SRT duration perception is affected by the saccadic response as well. However, we systematically provided feedback in an attempt to maintain an accurate calibration of the reported durations and reduce the impact of this effect. Moreover, the temporal relation between the temporal interval and saccade occurrence was fixed in our case so that the induced temporal distortion should not affect the overall psychophysics functions. Importantly, time perception has been found to be strongly related to motor action and planning^59–61^. For example, at the behavioral level, action development influences time perception accuracy^62^. At the neural level, time perception and motor control partially share the same neural network^59,60,63^ which includes the cerebellum^64^, Sensory Motor Area (SMA) and the cortico-thalamic-basal ganglia timing circuit (CTBGc)^65^. The scalar property, i.e. the linear relationship between the temporal variability and the mean of the interval, a well-known property of interval timing^34,66,67^, has been linked to neural activity in SMA^68^ as well as in the striatum^69,70^. Given the necessity of temporal coordination for motor action this strong relation is of functional importance and the ability to perceive our own motor reaction time is well in line with the notion that motor systems may access temporal information to regulate the timing of the action.

To our knowledge, our study is the first to provide evidence of the perception of one’s own saccadic reaction times. We suggest that such temporal information may be used by the saccadic system to adapt saccade triggering to the changing temporal properties of dynamic environments. Importantly, the limits of SRTs perception are likely to constrain the extent of temporal saccade control, a possibility that is absent from current decision models. Integrating these constraints might inform us regarding the specific mechanisms underlying the adaptation to the temporal regularities of the environment when foraging visual information.

## Methods

### General method

All experiments were conducted in two separate phases: a learning-phase (see further details in Supplementary Information) and a testing-phase using the method of constant stimuli.

In three different 2-AFC tasks (LD, AD and VD), participants had to estimate the duration of an interval in milliseconds by choosing a value between two options. In the LD experiment participants estimated their own saccade latency duration; in the AD experiment participants estimated the duration of a sound (a 500 Hz tone) and in the VD experiment the duration of a visual stimulus (a white rectangle). Following the stimulus (in AD and VD) or the movement (in LD), two numbers were displayed on the screen, one indicating the actual duration and the other one an incorrect duration.

Participants used a gamepad to select the value representing the perceived latency (in LD) or stimulus duration (in AD and VD). After the decision, the number turned green if the chosen value matched the actual duration and red if the chosen value was the incorrect one. In addition, a point (related to a monetary value of 10 cents) was given if five consecutive choices were correct and the total score was displayed every 50 trials in order to motivate the participant.

In the learning-phase, the difference between the correct and incorrect value was computed according to a set of percent-differences in a staircase starting from 50% and followed a decreasing function. Specifically, the percentage values used in the staircase were computed using the following function: $f (x)= {(1/2)}^{x}$. Where x ranged from 1 to 8 by steps of 0.125.

In the testing-phase we used a constant stimuli paradigm with 20 fixed percentage-difference, ranging from 50 to 2.5% by steps of 2.5%. A percent-difference was randomly selected in each trial. This phase consisted in three 220-trials sessions. A psychometric function was fitted to estimate the participant’s perceptive threshold at 75%. Pearson correlation coefficients were computed between the regression model coefficients against the perceptual thresholds.

In a fourth 2-AFC task (DT) only the two values were presented on the screen and the participants were asked to choose one of them. In this task the correct option was determined by the actual latency duration registered in the LD task; the incorrect option was represented by the same incorrect latency presented in the LD task.

### Latency duration perception task

Participants were seated in a dark and quiet room in front of a video monitor at a viewing distance of 60 cm. Eye movements were measured with an infrared video-based eye tracking system (Eyelink, SR Research Ltd.), sampled at 2000 Hz. Each trial started with a target, a small grey disk located on the left, 11º away from the centre of the screen. After a randomized variable delay ranging from 750 to 1250 ms (sampled from a uniform distribution) the target stepped rightward at a randomized amplitude (either 7, 10, 13, 16 or 19º) and a saccade toward the target was required. Both the fixation- and saccade-targets had a fixed 0.5º diameter and a 16 cd/m^2^ luminance against a dark grey background (luminance 1.78 cd/m^2^). In order to increase the dispersion in the latency distribution, the fixation-target was extinguished 140 ms after the saccade-target presentation, in an overlap paradigm^71,72^. Following the saccade offset, the saccade-target remained visible for a random interval ranging from 250 to 500 ms (sampled from a uniform distribution). If the saccade was not made properly, i.e. if there was an anticipation (latency shorter than 80 ms with respect to saccade-target onset), if the latency was longer than 500 ms, or the amplitude of the saccade was too short (amplitude less than 2°), the saccade-target was extinguished at saccade offset and the next trial started (i.e. the fixation-target reappeared on the left side of the screen).

### Visual stimulus duration task (VD)

A fixation target (0.5º in diameter) displayed for a randomized interval ranging from 750 to 1250 ms (sampled from a uniform distribution). The visual stimulus consisted of a rectangle with a base of 3.7º and height 0.7º. The durations of the stimulus corresponded to the duration of the participant’s saccades in the corresponding session rounded to the nearest 10 ms to match the 100 Hz refresh rate of the monitor. Both fixation points and visual stimulus had 16 cd/m^2^ luminance against a dark grey background with luminance 1.78 cd/m^2^.

## Participants

Twenty-four participants (7 males and 19 females) with no neurological or psychological impairments were tested in the four experiments. Participants S1 to S8, S17 and S21 (10 in total), performed the auditory task (AD); participants S9 to S24 (16 in total) performed the latency duration task (LD); eight of them, S17 to S24 also experienced the visual duration task (VD) and latency distribution estimation task (DT). Two of them where the authors (S17 and S21). Authors’ results did not systematically differ from the other participants’ ones. All participants had a normal or corrected-to-normal vision.

All experimental procedures received approval from the Ethical Committee in behavioural sciences of the University of Lille (Agreement n° 2015-2-S38) and conformed to the protocol set by Declaration of Helsinki. Participants were adequately informed regarding the entire procedure and we obtained written consent from all of them. At the end of the procedure, a full explanation was given in order to clarify any doubts and explain the experimental goals.

## Apparatus

In LD, VD and DT experiments, participants sat in a darkened room in front of a 22-in. IIYAMA HM204DT (1024 × 768 pixels at 100 Hz) CRT screen. Stimuli were generated and the experiments controlled by a computer using the Psychophysics Toolbox^73,74^ running Matlab (The Mathworks Inc., Natick, MA). Observers reported their choice using a gamepad.

In LD experiment, participants seat on an adjustable chair, the head and the chin rest on a support to minimize head movements, at 60 cm from the screen. Right Eye position was recorded by a Tower Eyelink 1000 sampling at 2000 Hz (SR Research Ltd, Osgoode, Ontario, Canada).

AD experiment was implemented on a Macintosh laptop with the Psychophysics Toolbox^73,74^ running in Matlab (The Mathworks Inc., Natick, MA) and the auditory stimuli were presented using headphones. Observers reported their choice pressing either the right or left arrow on the keyboard.

## Data analysis

### LD task: saccades analysis

In the LD task, a human observer validated each saccade manually; saccades with amplitude gain lower than 0.4 or larger than 1.5, or duration longer than 100 ms were discarded. In addition, saccades with latencies shorter than 80 ms or longer than 500 ms, or starting further than 2° away from the fixation-target position were discarded. We kept from 86 to 98% of all saccades across all participants.

### Method of constant stimuli: psychometric fit

In all experiments, a psychometric curve fitted the data collected in the three constant stimuli sessions. The proportion of correct responses against the corresponding percent-difference are modeled using a logistic function. To find the best fit in the 2AFC task we used three free parameters in the logistic function^75,76^:

1 $$\psi \left(x\right)=\frac{0.5+(1-0.5-\lambda )}{1+{e}^{-\frac{x-\alpha }{\beta }}}$$

where $\lambda$ is the lapse rate (represented by the difference between 100% correct responses and the function upper asymptote), $\alpha$ is the mean value in the distribution and $\beta$ is the slope. Because we used a 2AFC discrimination task, chance level would be at 50%. We therefore chose to use the conventional difference threshold at 75% as a discrimination criterion. To find the exact threshold corresponding to 75% we used the inverse function as followed:

2 $${\psi }^{-1}(.75)=-log\left(\frac{1-0.5-\lambda }{0.75-0.5}-1\right)*\beta +\alpha $$

The 95% confidence interval of the threshold was estimated using a bootstrap with 10,000 iterations.

## Supplementary information

Supplementary Information 1.

## Data Availability

The datasets generated during and/or analysed during the current study are available from the corresponding author on reasonable request.
