# Supplementary material for: Perception of saccadic reaction time
Source: Sci Rep. 2020 Oct 14;10:17192. doi: 10.1038/s41598-020-72659-3 (PMC7560701; doi:10.1038/s41598-020-72659-3)
Supplement: Supplementary file 1 — Supplementary Information 1. [file 41598_2020_72659_MOESM1_ESM.pdf]

PERCEPTION OF SACCADIC REACTION TIME

Valentina Vencato, Laurent Madelain

Supplementary Information

Supplementary methods

Staircase procedure

In the learning phases, we implemented an adaptive staircase procedure [1,2]. We implemented this adaptive psychophysical paradigm, i.e. a staircase or "up and down" procedure, to gradually train participants in a stimulus duration estimation. The difference between the two options (one representing the correct duration and the other representing the incorrect one) was gradually reduced based on the observer's performance. The incorrect option was computed based on the relative distance to the correct option according to specific percent-difference in a fixed staircase. The initial percent-difference was set to 50% and moved toward smaller values following a logarithmic proportion in a decreasing function. Specifically, the percent-differences in the staircase were computed using the following function: $f(x) = (1/2)^x$, where x is a number ranging from 1 to 8 by steps of 0.125. We used a 2-down-2-up rule such that two consecutive correct responses moved one step down toward a smaller percent-difference and two consecutive incorrect responses moved on step up toward a larger percent-difference. This up-down rule aimed at targeting the 50% performance level. The step size ratio Δ_-/Δ_+ (where Δ_- is the size of the step-down and Δ_+ is the size of the step up) was kept constant at around 0.92. This choice was based on the necessity to facilitate the convergence of the steps-sequence and have a larger resolution for smaller percent-differences. The percent-difference corresponding to the actual step position in the staircase was randomly either subtracted or added to the actual event duration to compute the incorrect option.

Data analysis

Learning.

The temporal discrimination learning has been quantified by computing the just noticeable difference, JND for each session of the learning phase of LD, VD and SD experiments in each participant. The JND [3] has been computed by averaging the percent-difference in the last four reversals (peaks and dips) of the staircase procedure (see Supplementary Fig. S1 online).

Supplementary Results

Learning.

Figure S1(B) plots the variations of JND in the learning sessions for each participant. In participants 12, 13, 16 and 19 the JND reached a plateau after 1, 4, 2 and 1 sessions respectively. The JND averaged across the last three learning sessions (where the plateau is reached) varies from 5%, for participants 12 and 19, to 10% for participants 13 and 16. The other participants did not exhibit a clear pattern in the changes of JND across sessions. Performance varies across sessions from a minimum of 2% to a maximum of 26% (see Supplementary Fig. S1(B) online). This variability might be due to inter-individual differences in sustained attention.

A correlation between JND in learning and the 75% threshold computed in the testing phase has been computed. Pearson's coefficient was not significant.

Saccadic distributions

Saccade latencies were quite variable across participants. Histograms of saccadic distributions show these inter-individual differences across participants (Supplementary Fig. S2 online). In the testing phase, median latencies ranged from 135ms to 253ms. The interquartile range, which quantifies the SRT dispersion within a distribution, were quite variable as well, ranging from 16ms up to 98ms.

Effect of target eccentricity

Two effects of target eccentricity have been reported that might affect the observers' estimate of their saccade latency. First, it has been shown that the eccentricity of a visual stimulus might affect its perceived duration [4]. Second, it is established that saccade latencies varies with the eccentricity of the target [5]. In the LD experiment we indeed found that latencies generally increased with target eccentricity from 154ms for the 7deg eccentricity to 187ms for the 19deg eccentricity (see Supplementary Fig. 3(A) online), although this trend was quite variable and not observed in 3 participants. Therefore, one might suppose that participants could have use the target eccentricity to report their saccade latency. This should induce a bias in the incorrect responses such that observers should choose more often a small incorrect value when eccentricity is small, and a large incorrect value when eccentricity is large. To probe this, we computed the frequency of choosing the small value for incorrect trials for each of the 5 target eccentricities. Results are plotted on Supplementary Fig. S3(B) online. It is apparent that there is no systematic bias as for the smallest amplitude the average proportion reaches 0.52 and 0.54 for the largest amplitude. It should also be noted that the bias is systematically below 0.5 for some participants and systematically above 0.5 for others. We compared the bias in the two smallest eccentricities (7 and 10 degrees) combined versus the ones in the two largest eccentricities (16 and 19 degrees) combined; the differences were not significant (sign test p-value=0.08). We are therefore confident that the target amplitude was not systematically used to report the perceived latencies.

Bibliography

1. Cornsweet, T. N. The Staircase-Method in Psychophysics. *Am. J. Psychol.* **75**, 485 (1962).
2. Treutwein, B. Adaptive psychophysical procedures. *Vision Res.* **35**, 2503–2522 (1995).
3. Meese, T. S. Using the standard staircase to measure the point of subjective equality: A guide based on computer simulations. *Percept. Psychophys.* **57**, 267–281 (1995).
4. Kliegl, K. M. & Huckauf, A. Perceived duration decreases with increasing eccentricity. *Acta Psychol. (Amst.)* **150**, 136–145 (2014).
5. Kalesnykas, R. P. & Hallett, P. E. Retinal eccentricity and the latency of eye saccades. *Vision Res.* **34**, 517–531 (1994).

SUPPLEMENTARY FIGURE LEGENDS

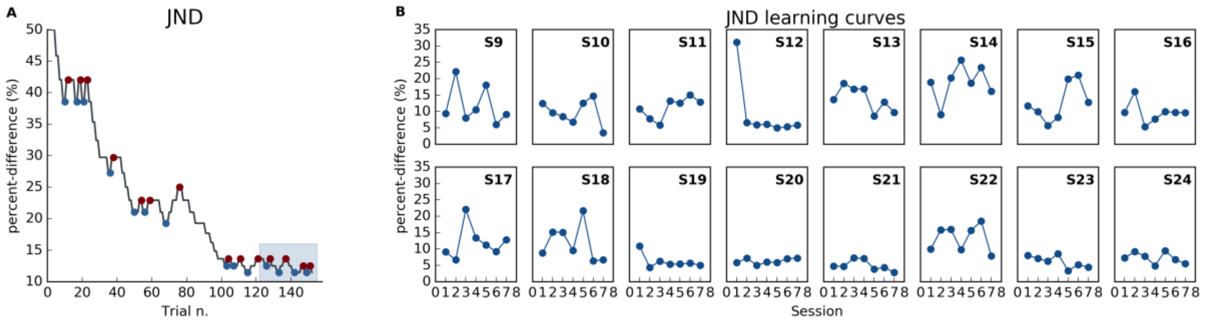

Figure S1

(A) Example of a trial-by-trial staircase in a learning-session in one participant: the percent-difference is plotted against the trial number. Dots represent the staircase reversals, red dots mark the percentage-difference increases, blue dots the percentage-difference decreases. The shaded area includes the last four peaks and the last four valleys that are then averaged to estimate the perceptual threshold at 50% i.e. the just-noticeable-difference.

(B) Perceptual threshold at 50% for each participant in the 7 learning sessions.

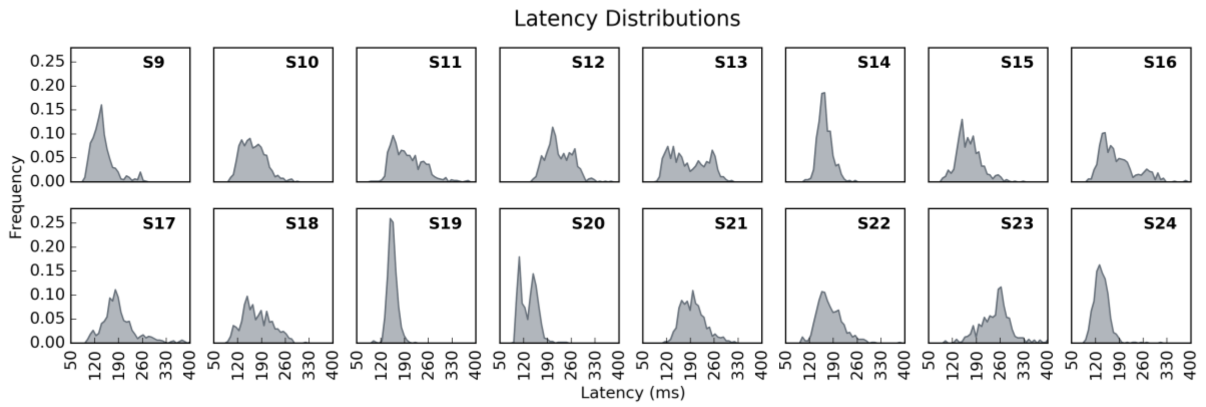

Figure S2

Saccade latency distributions for each one of the 16 participants in the test-phase sessions (method of constant stimuli). The latency frequency is plotted against the latency in milliseconds.

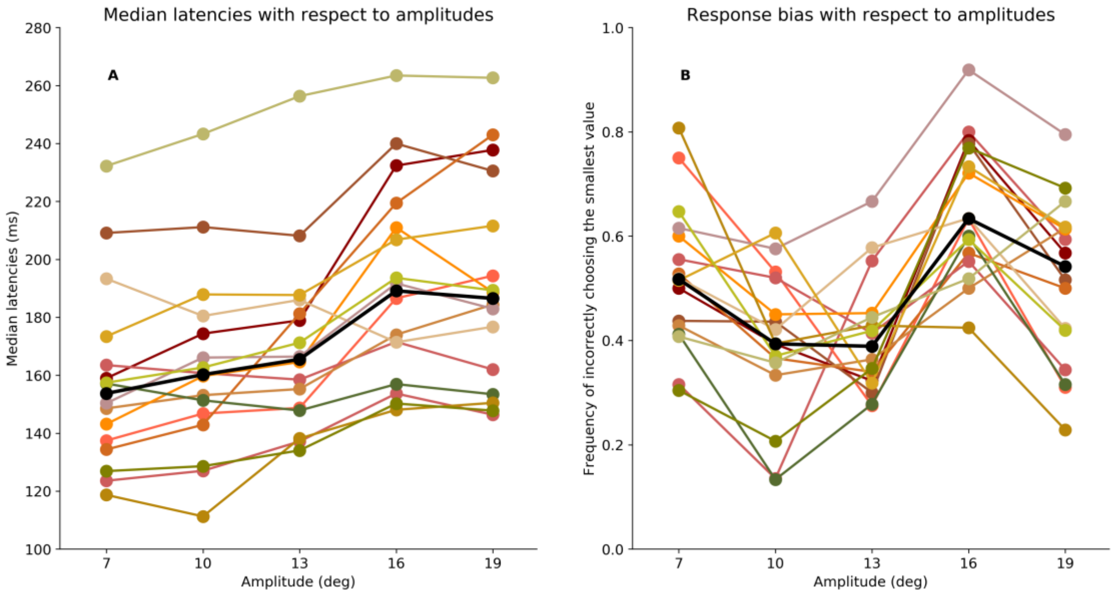

Figure S3

(A) SRTs medians plotted against the different degrees of amplitude for each participant. The black symbols plot the median latency across participants for each amplitude value.

(B) Bias expressed as the frequency of incorrectly choosing the smallest value plotted against the different degrees of amplitude for each participant. The black symbols plot the median frequency across participants for each amplitude value.
